# TGF-β1 modulates the homeostasis between MMPs and MMP inhibitors through p38 MAPK and ERK1/2 in highly invasive breast cancer cells

**DOI:** 10.1186/1471-2407-12-26

**Published:** 2012-01-19

**Authors:** Luciana R Gomes, Letícia F Terra, Rosângela AM Wailemann, Leticia Labriola, Mari C Sogayar

**Affiliations:** 1 Instituto de Química, Departamento de Bioquímica, NUCEL (Núcleo de Terapia Celular e Molecular), Universidade de São Paulo, São Paulo 05508-000, SP, Brazil

## Abstract

**Background:**

Metastasis is the main factor responsible for death in breast cancer patients. Matrix metalloproteinases (MMPs) and their inhibitors, known as tissue inhibitors of MMPs (TIMPs), and the membrane-associated MMP inhibitor (RECK), are essential for the metastatic process. We have previously shown a positive correlation between MMPs and their inhibitors expression during breast cancer progression; however, the molecular mechanisms underlying this coordinate regulation remain unknown. In this report, we investigated whether TGF-β1 could be a common regulator for MMPs, TIMPs and RECK in human breast cancer cell models.

**Methods:**

The mRNA expression levels of TGF-β isoforms and their receptors were analyzed by qRT-PCR in a panel of five human breast cancer cell lines displaying different degrees of invasiveness and metastatic potential. The highly invasive MDA-MB-231 cell line was treated with different concentrations of recombinant TGF-β1 and also with pharmacological inhibitors of p38 MAPK and ERK1/2. The migratory and invasive potential of these treated cells were examined in vitro by transwell assays.

**Results:**

In general, TGF-β2, TβRI and TβRII are over-expressed in more aggressive cells, except for TβRI, which was also highly expressed in ZR-75-1 cells. In addition, TGF-β1-treated MDA-MB-231 cells presented significantly increased mRNA expression of MMP-2, MMP-9, MMP-14, TIMP-2 and RECK. TGF-β1 also increased TIMP-2, MMP-2 and MMP-9 protein levels but downregulated RECK expression. Furthermore, we analyzed the involvement of p38 MAPK and ERK1/2, representing two well established Smad-independent pathways, in the proposed mechanism. Inhibition of p38MAPK blocked TGF-β1-increased mRNA expression of all MMPs and MMP inhibitors analyzed, and prevented TGF-β1 upregulation of TIMP-2 and MMP-2 proteins. Moreover, ERK1/2 inhibition increased RECK and prevented the TGF-β1 induction of pro-MMP-9 and TIMP-2 proteins. TGF-β1-enhanced migration and invasion capacities were blocked by p38MAPK, ERK1/2 and MMP inhibitors.

**Conclusion:**

Altogether, our results support that TGF-β1 modulates the mRNA and protein levels of MMPs (MMP-2 and MMP-9) as much as their inhibitors (TIMP-2 and RECK). Therefore, this cytokine plays a crucial role in breast cancer progression by modulating key elements of ECM homeostasis control. Thus, although the complexity of this signaling network, TGF-β1 still remains a promising target for breast cancer treatment.

## Background

Breast cancer is a worldwide health problem for women, since it is the first in incidence and the second in mortality among cancer types [1]. Similarly to the majority of solid tumors, the main death factor attributed to breast cancer is the process of cell spreading (metastasis) from primary tumor to secondary sites [2]. The metastatic process involves a complex cascade of events, including the organized breakdown of the extracellular matrix (ECM) [3-5]. Matrix metalloproteinases (MMPs) and their specific inhibitors, known as tissue inhibitors of MMPs (TIMPs) and the membrane-associated MMP inhibitor (RECK), are essential regulators of ECM degradation [6-9].

The MMPs constitute a large family of endopeptidases, which are responsible for degrading almost all ECM components, with each ECM element being cleaved by a specific MMP or a set of MMPs [10]. Consistent with their role in tumor progression, high levels of several MMP family members have been shown to correlate with poor prognosis [11,12]. Among the several MMPs previously related to breast cancer progression, the gelatinases (MMP-2 and MMP-9) stand out for their collagen type IV specific degradation capacity, in view of the fact that it is an abundant ECM component [13,14]. In association with TIMP-2, MMP-14 is involved in MMP-2 activation, being also correlated with breast cancer progression [15]. Given that ECM proteolysis is related to important physiological and pathological processes, homeostasis of the ECM degradation is tightly controlled by the balance between MMPs and MMP inhibitors [6-9].

Together, the secreted tissue inhibitors of MMPs (TIMPs) are able to reversibly inhibit the activity of all MMPs family members. Although first described as anti-invasive molecules, high levels of TIMP-1, TIMP-2 and TIMP-4 [12,16,17] have been associated to adverse prognostic and cellular aggressiveness in breast tumors. This apparently controversial expression profile of TIMPs could be the result of their recently described role as multifunctional molecules [8]. The membrane-associated MMP inhibitor, RECK (reversion-inducing cysteine-rich protein with Kazal motifs), is able to suppress tumor invasion and metastasis by negatively regulating MMP-2, MMP-9 and MMP-14 [9,18,19]. As reviewed by Noda and Takahashi [19], RECK is described as a good prognosis marker, and several prior reports have demonstrated that RECK expression is decreased during cancer progression [9,19]. However, its role in breast cancer remains unclear, since no functional analysis of the RECK gene is yet available for this model. Moreover, unlike other cancer types, previous results from our laboratory showed that RECK transcript levels are higher in highly invasive and metastatic cell lines compared to less aggressive breast cell lines [12].

We have previously shown a significantly positive correlation between the mRNA expression levels of MMPs, TIMPs and RECK, both in cell line models as well as in tumor tissue samples [12], suggesting that the expression of these molecules, at least at the transcriptional level, may be regulated by common factors and signaling pathways in breast cancer. Like that of MMPs and their inhibitors, a high expression of TGF-β1 (Transforming growth factor-β 1) has been positively correlated with metastasis and tumor aggressiveness in mammary models [11]. Because TGF-β1 has been shown to be involved in mechanisms regulating the expression and activity of some MMPs and/or MMP inhibitors in different models, [20-28], this cytokine seemed to be an interesting candidate to be tested as a common modulator of both types of molecules.

TGF-β is a multifunctional cytokine, which modulates a wide variety of biological processes, including cell growth, differentiation, apoptosis, immunity, extracellular matrix production, angiogenesis, migration and invasion [29,30]. However, TGF-β may induce entirely different cellular responses, depending on the cell type and stimulation context, both under physiological and pathological conditions [29,31]. Similarly, the role of TGF-β in cancer progression has been shown to be multifaceted, given that this cytokine acts as a potent growth inhibitor, as an inducer of EMT (epithelial-mesenchymal transition) as well as a metastasis inducer, depending on the tumor stage [32-34]. TGF-β isoforms (TGF-β1, TGF-β2 and TGF-β3) signal after binding to their transmembrane serine/threonine kinase receptor type II (TβRII), followed by association and trans-phosphorylation of TGF-β receptor type I (TβRI). In addition to the classical TGF-β-induced signal transduction by Smads, it is well known that this cytokine also signals in a Smad-independent manner, by induction of other pathways, such as the extracellular signal-regulated kinase 1/2 (ERK1/2) and the p38 MAP kinase (p38 MAPK) [35]. Previous reports have shown the direct function of these MAPK pathways in signal transduction of TGF-β-modulated cellular migration and invasion [21,35].

In the present study, we investigated the role of TGF-β1 as a common regulator for MMPs, TIMPs and RECK in highly invasive human breast cancer cells and the involvement of the ERK1/2 and p38 MAPK pathways in this mechanism.

## Methods

### Reagents

The recombinant TGF-β1 and the neutralizing antibody anti-TGF-β1 were from R&D Systems (Minneapolis, MN, USA). Antibodies against MMP-14 (113-5B7), TIMP-1 (7-6 C), TIMP-2 (67-4H11) and T1MP-3 (136-13H4) were purchased from Merck (Darmstadt, Germany). Antibodies against p-ERK1/2, GAPDH and β-Tubulin were obtained from Santa Cruz (Santa Cruz, CA, USA). The antibodies against p-p38 MAPK, total ERK1/2, total p38 MAPK and RECK were purchased from Cell Signaling (Beverly, MA, USA). The pharmacological inhibitors against p38 MAPK (SB203680) and ERK1/2 (PD98059) were obtained from Tocris Bioscience (Bristol, UK). The broad-spectrum MMP inhibitor (GM6001) was purchased from Millipore (Billerica, MA, USA).

### Cell lines and culture conditions

Five human breast cancer cell lines displaying different degrees of invasiveness and metastatic potential were used in this study [12]. The MCF-7 and Hs578T cell lines were maintained in phenol red-free Dulbecco's Modified Eagle Medium (LGC Biotecnologia, Cotia, Brazil) supplemented with fetal bovine serum (Cultilab, Campinas, Brazil) to a final concentration of 10%. The ZR-75-1, MDA-MB-231 and MDA-MB-435 were cultured in RPMI medium without phenol red (LGC Biotecnologia) supplemented with 10% fetal bovine serum. For MMPs and MMP inhibitors mRNA analysis by qRT-PCR, total RNA was extracted when these cells achieved 80-90% confluence. For TGF-β1 treatment, the MDA-MB-231 cells were plated in serum-containing medium and then serum-starved in a final concentration of 0.1% overnight prior to treatment with TGF-β1 (10 ng/mL). In the "loss of function" study these cells were treated with different concentration of anti-TGF-β1 antibody, being that the range of tested concentrations (1, 10, 25 or 50 ng/mL) include those recommended by the manufacturer. The ERK1/2 or p38 MAPKs inhibitors were added 1 h prior to TGF-β1 treatment. The MDA-MB-231 cells were treated with TGF-β1 for 20 h.

### Quantitative RT-PCR studies

Total RNA from cell lines cultured and treated as described above was extracted using the RNAspin Mini Kit (GE Healthcare, Waukesha, WI, USA). For cDNA synthesis, 1 μg of total RNA was reverse-transcribed using oligo-dT primers and the Superscript Amplification System (Life Technologies, Carlsbad, CA, USA). Quantitative RT-PCR was carried out using SYBR Green PCR Master Mix (Life Technologies). Table 1 shows the primers used, with the optimal concentration (in the range of 100 nM to 600 nM). The cycling conditions were 50°C for 2 min, 95°C for 10 min, followed by 40 cycles of 95°C for 15 s and 60°C for 30 s. The mRNA expression levels of GAPDH, HPRT and H-MBS genes were subjected to the GeNorm computational program analysis [36]. The HPRT and H-MBS transcriptional expression levels, classified as the two most stable genes according to GeNorm analysis, were used to calculate the GeNorm Normalization Factor used as the endogenous control for the qRT-PCR. The amplification efficiency analyzed was calculated for each gene from the given slope in a linear regression curve of Ct values versus log of cDNA concentration. The corresponding PCR efficiency (E) of one cycle in the exponential phase was calculated according to the equation: E = 10^[-1/slope]^. Relative expression levels were calculated according to the Pfaffl model [37].

**Table 1:** Sequence of primers used

Gene	Oligonucleotide sequence (5'-3')
MMP-2	F: AGCTCCCGGAAAAGATTGATG
	R: CAGGGTGCTGGCTGAGTAGAT
MMP-9	F: CACGCACGACGTCTTCCA
	R: AAGCGGTCCTGGCAGAAAT
MMP-14	F: GCAGAAGTTTTACGGCTTGCA
	R: TCGAACATTGGCCTTGATCTC
TIMP-1	F: CCGCAGCGAGGAGTTTCTC
	R: GAGCTAAGCTCAGGCTGTTCCA
TIMP-2	F: CGACATTTATGGCAACCCTATCA
	R: GGGCCGTGTAGATAAACTCTATATCC
TIMP-3	F: ATCACCTGGGTTGTAACTGCAA
	R: CGCTCCAGAGACACTCGTTCTT
RECK	F: TGCAAGCAGGCATCTTCAAA
	R: ACCGAGCCCATTTCATTTCTG
TGF-β1	F: GGCCCTGCCCCTACATTT
	R: CCGGGTTATGCTGGTTGTACA
TGF-β2	F: TCAAGAGGGATCTAGGGTGGAA
	R: GGCARGCTCCAGCACAGAA
TGF-β3	F: CAGCTCTAAGCGGAATGAGCAG
	R: TATAGCGCTGTTTGGCAATGTG
TβRI	F: AAGTCATCACCTGGCCTTGGT
	R: TGCGGTTGTGGCAGATATAGAC
TβRII	F: AATATCCTCTGAAGAACGACCTAA
	R: TCCCACCTGCCCACTGTTA
PAI-1	F: GGCTGACTTCACGCGTCTTTCAG
	R: GTTCACCTCGATCTTCACTTTCTG
GAPDH	F: ACCCACTCCTCCACCTTTGA
	R: CTGTTGCTGTAGCCAAATTCGT
H-MBS	F: TGGACCTGGTTGTTCACTCCTT
	R: CAACAGCATCATGAGGGTTTTC
HPRT	F: TCATTATGCTGAGGATTTGGAAAG
	R: GGCCTCCCATCTCCTTCATC

### Western blotting

Cultures were washed with ice-cold PBS and then lysed with lysis buffer (50 mM Tris, pH 7.5; 300 mM NaCl and 5 mM EDTA, pH 8.0) supplemented with 1× protease inhibitor mix (GE Healthcare). For protein phosphorylation analysis, a phosphatase inhibitor mix (GE Healthcare) was also added. The homogenate was centrifuged for 30 min at 12,000 × g and the supernatant fraction was then collected and stored at -70°C. Conditioned medium was concentrated using Centricon Centrifugal Filters (Millipore). The total protein content for each sample was quantified using a Bio-Rad kit (Bio-Rad, Hercules, CA, USA). Equal amounts (50-100 μg) of proteins from each extract were boiled in Laemmli's sample buffer containing 5% β-mercaptoethanol for denaturation. The protein samples were fractionated by SDS-PAGE and then electro-transferred to nitrocellulose membranes, which were blocked and then incubated for 2 h at room temperature or overnight at 4°C, depending on the antibody. Immunoreactive proteins were detected with an appropriate secondary horseradish peroxidase-coupled antibody (Vector, Burlingame, CA, USA) and visualized using ECL Western blot reagent (GE Healthcare). Quantitative densitometry of the electrophoretic bands images was carried out with the ImageQuant 5.2 software (GE Healthcare).

### Gelatin zymaography assays

Gelatin zymography of conditioned medium was used to observe the levels of MMP-2 and MMP-9 produced by MDA-MB-231 cell lines treated with TGF-β1 and/or MAPK inhibitors. These samples were separated in a 10% SDS-polyacrylamide gel electrophoresis co-polymerized with the enzyme substrate, 0.1% denatured type I collagen (gelatin type A, Sigma, St. Louis, MO, USA), [38]. After eletrophoresis, the gels were washed at room temperature with 2.5% Triton X-100 in water for 1 h under orbital shaking. The washed gels were incubated for 48 h at 37°C in substrate buffer containing 50 mM Tris buffer (pH 8.5) and 10 mM CaCl_2 _and then stained with Coomassie Blue R-250 (Sigma) and destained with 40% Methanol (Merck), 10% Acetic Acid (Merck) in water. Gelatinolytic activity was visualized as negative staining bands, the image was inverted and the intensity of each band was normalized to the number of cells. Each independent experiment was performed in duplicate.

### Migration and invasion assays

1 × 10^4 ^MDA-MB-231 cells were plated in the top chambers of 8 μm pore transwells (BD, Franklin Lakes, NJ, USA) in a low serum medium and pre-treated for 1 h with PD98059, SB203680 or GM6001. After this period of pre-treatment the medium at the bottom chamber was supplemented with 10 ng/mL of TGF-β1. These cells were allowed to migrate towards medium contained this cytokine over a period of 8 h. To assess the invasive potential of this cell line, the same protocol as above described was used with matrigel-coated transwells (BD Biosciences). In the invasion assays the cells were allowed to invade for 24 h. Upon this period of time, cells at the top chamber were removed and the cells at the bottom of the filter were stained and fixed with Coomassie Blue 0.125% in methanol: acetic acid:H_2_O (45:10:45, v/v/v) for 15 min. The number of cells per filter was counted on images from Nikon microscope using 10× objective lens. Duplicate wells were used per condition in each independent experiment.

### Statistical analysis

All statistical analyses were performed using the GraphPad Prism 5.0 program. Results are presented as mean ± standard deviation. Statistical significance was determined using the nonparametric KrusKal-Wallis test and the Dunns post test. Statistically significant differences were considered when *p *< 0.05. One way ANOVA variance analysis and Tukey-Kramer test were employed to calculate *p*-values in migration and invasion assays.

## Results

### Aggressiveness of breast cancer cell lines correlates with the expression levels of the MMPs and their inhibitors and with the TGF-β isoforms and receptors

**Figure 1:**
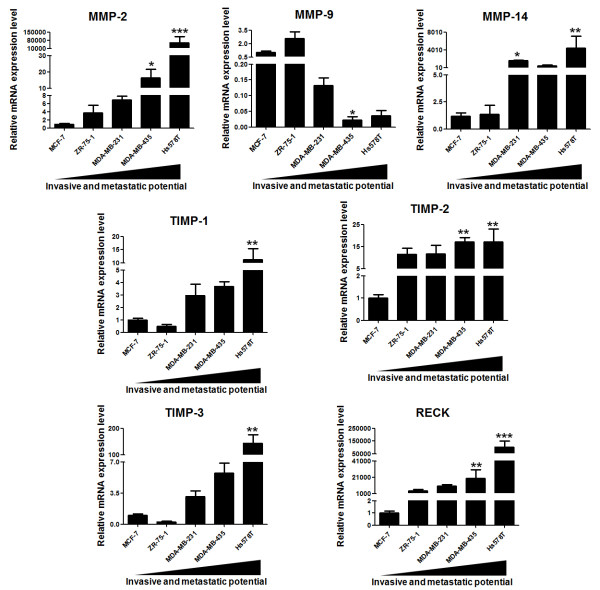
**Relative mRNA expression levels of MMPs and MMPs inhibitors in breast cancer cell lines**. Total RNA from a panel of five human breast cancer cell lines displaying different invasion and metastatic potentials were used to analyze the mRNA expression levels of MMPs (MMP-2, MMP-9 and MMP-14) and MMPs inhibitors (TIMP-1, TIMP-2, TIMP-3 and RECK) by qRT-PCR. Results are presented as means ± standard errors from three independent experiments, each measured in triplicate. *, *p *< 0.05, **, *p *< 0.01 and *** *p *< 0.001, all versus control (MCF-7).

Previous results from our laboratory indicated a positive correlation between high mRNA expression levels of MMPs and their inhibitors with breast cancer progression, both in cellular models and in tumor tissue samples, with all five human breast cancer cell lines displaying different invasive and metastatic potential when maintained in culture for 3 or 5 days [12]. Since these cell lines display distinct growth rates upon the same time in culture, they end up achieving different confluence levels. Bachmeier and collaborators demonstrated that MMPs and MMP inhibitors are differentially expressed at distinct cellular densities [39]. This report showed that the mRNA expression levels of MMP-2, MMP-9, TIMP-1 and TIMP-2 are modulated by the percentage of cell confluence in the breast cancer cell lines, including MCF-7 and MDA-MB-231 [39]. For this reason, we first analyzed the mRNA expression levels of MMP-2, MMP-9, MMP-14, TIMP-1, TIMP-2, TIMP-3 and RECK, in the same panel of five human breast cancer cell lines, but now maintained in culture until achieving 80-90% confluence. The relative mRNA expression levels of MMP-2, MMP-14, TIMP-1, TIMP-2, TIMP-3 and RECK were, in general, higher in highly invasive and metastatic cell lines (MDA-MB-231, MDA-MB-435 and Hs578T), when compared to less aggressive ones (MCF-7 and ZR-75-1) (Figure 1). The mRNA expression levels of MMP-2 were significantly elevated (*p *< 0.05) in the MDA-MB-435 and in the Hs578T (*p *< 0.001) breast cancer cell lines relative to MCF-7 cells. Similarly, MMP-14 mRNA was significantly overexpressed in highly aggressive cells, such as MDA-MB-231 (*p *< 0.05) and Hs578T (*p *< 0.01) cells. The most invasive and metastatic cell line, Hs578T, displayed significantly higher mRNA expression levels of TIMP-1 (*p *< 0.001) and TIMP-3 (*p *< 0.001) than the MCF-7 cell line. The expression of TIMP-2 was significantly higher (*p *< 0.01) in the most aggressive cell lines MDA-MB-435 and Hs578T, when compared with the least invasive one (MCF-7). Unlike other MMPs and MMP inhibitors, the expression profile of MMP-9 presented an opposite pattern since its transcriptional levels were significantly lower (*p *< 0.05) in MDA-MB-435 cells as compared to MCF-7 (Figure 1).

**Figure 2:**
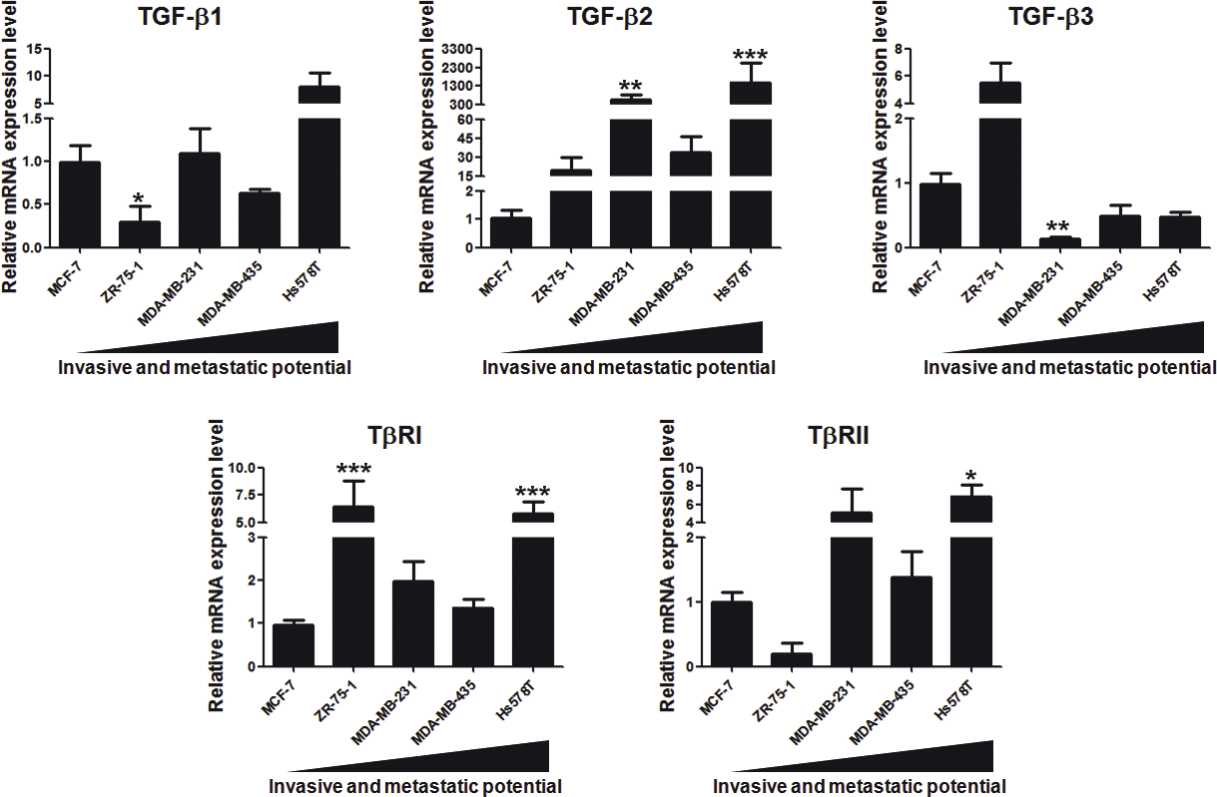
**Relative mRNA expression levels of TGF-β isoforms and receptors in breast cancer cell lines**. Total RNA from a panel of five human breast cancer cell lines displaying different invasion and metastatic potentiasl were used to analyze the mRNA expression levels of TGF-β isoforms (TGF-β1, TGF-β2 and TGF-β3) and TGF-β receptors (TβRI and TβRII) by qRT-PCR. Results are presented as means ± standard errors from three independent experiments, each measured in triplicate. *, *p *< 0.05, **, *p *< 0.01 and *** *p *< 0.001, all versus control (MCF-7).

In order to analyze whether TGF-β could act as a common regulator of MMPs, TIMPs and RECK in human breast cancer cell models, we investigated whether these cellular models express key members of the TGF-β network. Thus, we analyzed the mRNA expression levels of TGF-β isoforms (TGF-β1, TGF-β2 and TGF-β3) and their receptors (TβRI and TβRII) by qRT-PCR in this panel of five human breast cancer cell lines in cultures that had reached the same confluence level (Figure 2). Our results demonstrate that TGF-β2 is significantly overexpressed in MDA-MB-231 (*p *< 0.01) and Hs579T (*p *< 0.001) cell lines relative to MCF-7. Similarly, the TGF-β receptors, TβRI and TβRII, were highly expressed in the most aggressive cell line Hs578T (*p *< 0.001 and *p *< 0.05, respectively). In contrast, the mRNA levels of TGF-β3 were significantly lower (*p *< 0.01) in the highly invasive MDA-MB-231 cell line relative to the least aggressive one (MCF-7). The TGF-β1 transcriptional level was lower (*p *< 0.05) in ZR-75-1 cells than in MCF-7. Thus, these TGF-β pathway members are expressed by the cell lines included in this human breast cancer cell panel. These data also suggest that, following the same tendency as that of MMPs, TIMPs and RECK, the transcriptional levels of some TGF-β isoforms and receptors are partially correlated with cellular aggressiveness.

### TGF-β1 induces coordinate expression of MMP-2, MMP-9 and TIMP-2 in MDA-MB-231 breast cancer cells, but inhibits RECK protein expression levels

**Figure 3:**
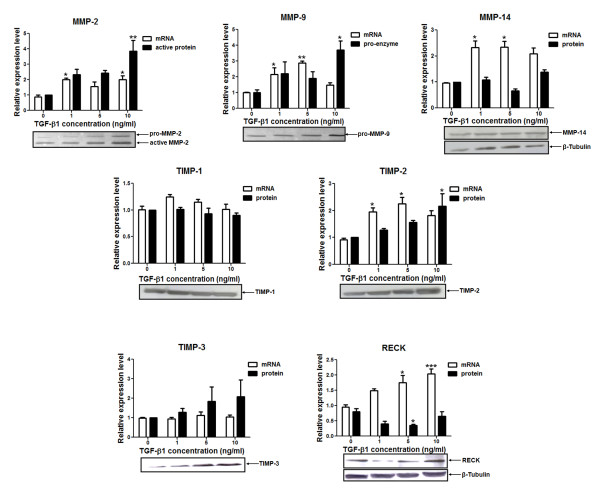
**Relative mRNA and protein expression levels of MMPs and MMPs inhibitors in the MDA-MB-231 cell line treated with different concentrations of TGF-β1**. The mRNA expression levels of MMPs (MMP-2, MMP-9 and MMP-14) and MMPs inhibitors (TIMP-1, TIMP-2, TIMP-3 and RECK) were analyzed by qRT-PCR using the total RNA from the MDA-MB-231 cells treated with 0, 1, 5 or 10 ng/mL TGF-β1 for 20 h. The levels of pro-enzyme and active MMP-2 and MMP-9 proteins were evaluated by zymography. Total protein lysates were used to measure the protein expression levels of MMP-14 and RECK by Western Blotting. The β-tubulin levels were used as protein loading control for Western Blot analysis. Conditioned medium was used to analyze the protein levels of TIMPs (TIMP-1, TIMP-2 and TIMP-3) by Western blotting. The results are presented in graphics as means ± standard errors from three independent experiments. The Western-blotting figures are representative of one experiment. *, *p *< 0.05, **, *p *< 0.01 and *** *p *< 0.001, all versus control (untreated cell).

Cancer cells with different aggressiveness respond to TGF-β1 treatment in distinct ways. In general, this cytokine plays a role as an invasion, EMT and metastasis inducer in advanced tumors [40,41]. Thus, in order to analyze the role of TGF-β1 as a common regulator of the MMPs and their inhibitors in a breast cancer cell model, we treated the highly invasive MDA-MB-231 cell line with different concentrations (0, 1, 5 or 10 ng/mL) of recombinant TGF-β1 for 20 h. The mRNA expression levels of PAI-I, a well-known TGF-β1 transcriptional target, was used as a positive control for the MDA-MB-231 treatment with this cytokine. As expected, we found a greater than 10-fold increase in PAI-I expression in TGF-β1-treated cells relative to untreated controls (Additional file 1) for all TGF-β1 concentrations tested, confirming that this cell line was still responsive to TGF-β1 treatment. Upon treatment with TGF-β1, the MDA-MB-231 cell line showed significantly increased mRNA expression levels of MMPs (MMP-2, MMP-9 and MMP-14) and MMP inhibitors (TIMP-2 and RECK) (Figure 3). The mRNA expression of MMP-2 was significantly upregulated in MDA-MB-231 cells upon treatment with 1 ng/mL (*p *< 0.05) and 10 ng/mL (*p *< 0.05) of TGF-β1, relative to the untreated control cultures. Statistically significant increased transcriptional expression levels of MMP-9 were verified upon treatment of these cells with 1 ng/mL (*p *< 0.05) and 5 ng/mL (*p *< 0.01) of recombinant TGF-β1. The MMP-14 mRNA levels were also significantly increased in the MDA-MB-231 cells upon treatment with 1 ng/mL (*p *< 0.05) and 10 ng/mL (*p *< 0.05) of TGF-β1. The mRNA expression levels of the MMP inhibitors were also upregulated in TGF-β1-treated MDA-MB-231 cells (Figure 3). TIMP-2 expression levels were higher in MDA-MB-231 cells treated with 1 ng/mL (*p *< 0.05) and 5 ng/mL (*p *< 0.05) of TGF-β1 than in the untreated ones. Similarly, cells treated with 5 ng/mL (*p *< 0.05) and 10 ng/mL (*p *< 0.001) of this cytokine displayed higher RECK mRNA levels than untreated cultures (Figure 3).

The treatment with recombinant TGF-β1 was also able to increase the protein levels of MMP-2, MMP-9 and TIMP-2, but downregulated RECK protein levels (Figure 3). By Zymography assays, we verified that the active MMP-2 (*p *< 0.01) and the pro-enzyme MMP-9 (*p *< 0.05) levels were significantly increased in MDA-MB-231 upon treatment with 10 ng/mL of TGF-β1, relative to the untreated condition. Like MMPs, TIMP-2 protein levels were also significantly (*p *< 0.05) increased in MDA-MB-231 cells treated with the highest TGF-β1 concentration tested. Conversely, RECK protein levels were decreased in TGF-β1-treated MDA-MB-231 cells. This TGF-β1-mediated downregulation of RECK protein levels was statistically significant at 5 ng/mL (*p *< 0.05) treatment conditions (Figure 3). Altogether, these results support that TGF-β1 modulates the mRNA and protein levels of MMPs (MMP-2 and MMP-9) as much as their inhibitors (TIMP-2 and RECK) in a dose-dependent manner.

**Figure 4:**
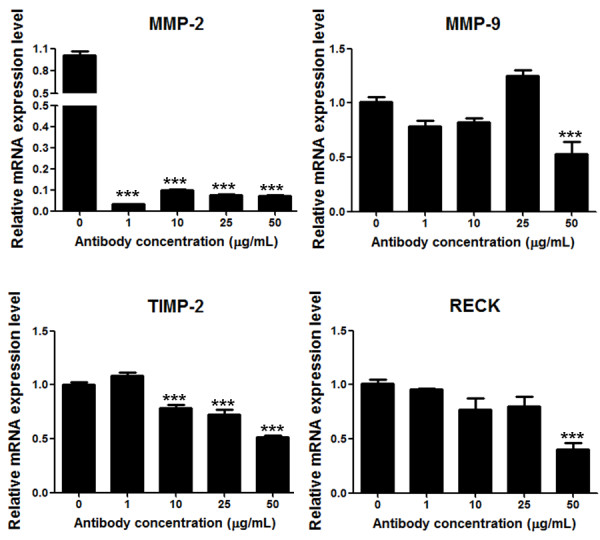
**Analysis of the effect of TGF-β1 loss of function on the expression levels of MMPs and MMPs inhibitors by the MDA-MB-231 cell line upon treatment with a neutralizing anti-TGF-β1 antibody**. The mRNA expression levels of MMPs (MMP-2, MMP-9) and MMPs inhibitors (TIMP-2 and RECK) were analyzed by qRT-PCR using total RNA from MDA-MB-231 cells treated for 24 h with 0, 1, 10, 25 or 50 ng/mL of a specific neutralizing antibody to the endogenous TGF-β1 bioactivity. The results are presented as means ± standard errors from two independent experiments. **, *p *< 0.01 and *** *p *< 0.001, all versus control (untreated cell).

In order to obtain direct evidence of the role of TGF-β1 on modulation of the expression of MMPs and their inhibitors, a "loss of function" study was pursued. To this end, the endogenous TGF-β1 activity of the MDA-MB-231 cell line was inhibited by using a specific antibody for neutralization of this cytokine. The MDA-MB-231 cells were treated with different concentrations (0, 1, 10, 25 or 50 ng/mL) of anti-TGF-β1 antibody for 24 h. As shown in the Additional File 1, the efficiency of TGF-β1 activity blockage was confirmed, since the mRNA levels of PAI-I, a well known TGF-β1 target, significantly decreased (*p *< 0.01) in cells subjected to higher antibody concentrations (25 and 50 ng/mL). Subsequently, the effect of TGF-β1 inhibition in the expression levels of MMPs and MMP inhibitors was assessed. The results, shown in Figure 4, demonstrated that treatment with the anti-TGF-β1 antibody was able to significantly inhibit (*p *< 0.001) the mRNA expression levels of MMP-2, MMP-9, TIMP-2 and RECK in MDA-MB-231 cells.

### TGF-β1 induces ERK1/2 and p38 MAPK phosphorylation with distinct kinetics

**Figure 5:**
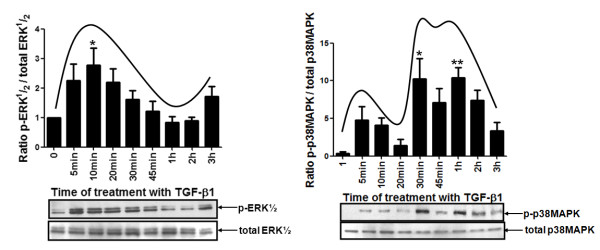
**Kinetics profile of phospho-ERK1/2 and phospho-p38 MAPK proteins in MDA-MB-231 cell line treated with TGF-β1**. Total protein lysates from the MDA-MB-231 cells treated with 10 ng/mL of TGF-β1 for different periods of time (0, 5 min, 10 min, 20 min, 30 min, 45 min, 1 h, 2 h and 3 h) were used to analyze the protein levels of total and phosphorylated forms of ERK1/2 and p38 MAPK by Western blotting. These results were analyzed and used to calculate the p-ERK1/2/total ERK1/2 and p-p38 MAPK/total p38 MAPK ratios during the kinetic of MDA-MB-231 treatment with TGF-β1. The results are presented in graphics as means ± standard errors from three independent experiments. The Western blotting figures are representative of one experiment. *, *p *< 0.05, ** and *p *< 0.01, all versus control (untreated cell).

To explore the role of ERK1/2 and p38 MAPK pathways in this proposed mechanism, we tested whether TGF-β1 is able to induce phosphorylation of these signal transduction proteins. Total protein extracts were obtained from MDA-MB-231 cells treated with 10 ng/mL of TGF-β1 for different periods of time (0, 5 min, 10 min, 20 min, 30 min, 45 min, 1 h, 2 h and 3 h) and the levels of ERK1/2 and p38 MAPK activation were analyzed by Western Blotting. As shown in Figure 5, TGF-β1 treatment induced a significant phosphorylation (*p *< 0.05) of both ERK1/2 as well as p38 MAPK. We also observed that these MAPKs showed two activation peaks. The first one was reached shortly after TGF-β1 addition (10 min and 30 min for p-ERK1/2 and p-p38 MAPK, respectively) while the second one was achieved after longer periods of time of treatment with this cytokine (1 h for p-p38 MAPK).

### Inhibition of ERK1/2 blocks TGF-β1-mediated upregulation of MMP-9 and TIMP-2 and increases the level of RECK protein

**Figure 6:**
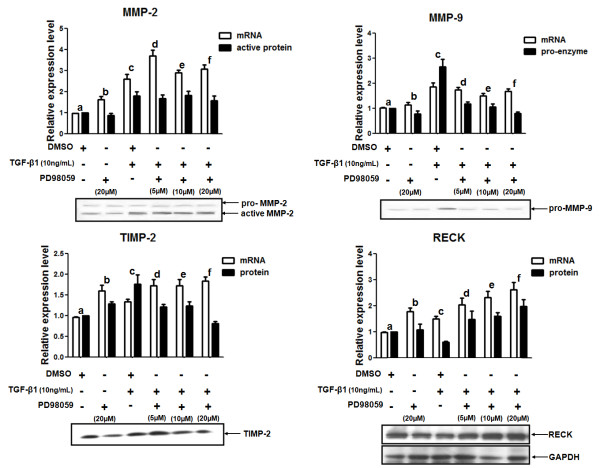
**Relative mRNA and protein expression of MMPs and MMPs inhibitors in MDA-MB-231 cells treated with TGF-β1 and an ERK1/2 inhibitor**. MDA-MB-231 cells were pre-treated for 1 h with different concentrations (0, 5, 10 or 20 μM) of PD98059 (ERK1/2 pharmacological inhibitor) and then stimulated with 10 ng/mL TGF-β1 by 20 h. Total RNA from these samples was used to analyze the mRNA expression levels of MMPs (MMP-2 and MMP-9) and MMPs inhibitors (TIMP-2 and RECK) by qRT-PCR. The levels of pro-enzyme and active MMP-2 and MMP-9 proteins were evaluated by zymography. Total protein lysates were used to measure the protein expression levels of RECK by Western blotting. The GAPDH protein expression was used as the loading control in Western blotting assays. Conditioned medium from these cultures were also utilized to analyze the TIMP-2 protein levels by Western blotting. Results are presented in graphics as means ± standard errors from three independent experiments. The Western blotting figures are representative of one experiment. For mRNA expression levels fold change: MMP-2 (a versus c: *p *< 0.05, a versus d: *p *< 0.01, a versus e, f: *p *< 0.001), MMP-9 (a versus c, d, f: *p *< 0.01), TIMP-2 (a versus b: *p *< 0.01, a versus c: *p *< 0.05, a versus d, e, f: *p *< 0.001) and RECK (a versus b, c: p < 0.05, a versus d, e, f: p < 0.001). For protein expression levels fold change: MMP-2 (a versus c: *p *< 0.01, a versus d, e: *p *< 0.05), MMP-9 (a versus c: *p *< 0.001, c versus f: *p *< 0.001), TIMP-2 (a versus c: *p *< 0.01, c versus f: *p *< 0.001) and RECK (a versus c: *p *< 0.01, a versus f: *p *< 0.05, c versus e, f:: *p *< 0.001).

The role of ERK1/2 pathway in TGF-β1-mediated regulation of MMPs and MMP inhibitors was also evaluated. Different concentrations (0, 5, 10 or 20 μM) of an ERK1/2 pharmacological inhibitor (PD98059) were used to pre-treat MDA-MB-231 cells for 1 h. These cultures were further stimulated with 10 ng/mL of TGF-β1 for 20 h. By qRT-PCR, we found that the ERK1/2 inhibitor did not affect the TGF-β1-mediated induction of MMP-2, MMP-9, TIMP-2 and RECK mRNA expression mediated by TGF-β1 treatment (Figure 6). However, the highest concentration of PD98059 significantly decreased (*p *< 0.001) the amount of MMP-9 and TIMP-2 protein levels following TGF-β1 treatment. ERK1/2 inhibition not only blocked the TGF-β1-mediated downregulation of RECK protein production, but also significantly increased RECK mRNA expression. Cells treated with 20 μM of PD98059 and 10 ng/mL of TGF-β1 presented significantly higher expression of RECK relative to cells treated with vehicle (*p *< 0.05) or with TGF-β1 only (*p *< 0.001) (Figure 6). These results suggest that the ERK1/2 activity is essential for the modulation of MMP-9, TIMP-2 and RECK expression by TGF-β1.

### p38 MAPK inhibition blocked the TGF-β1-mediated increase in MMP-2 and TIMP-2 protein levels

**Figure 7:**
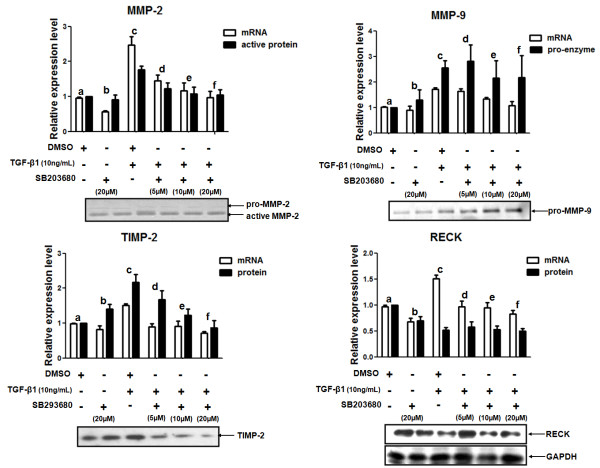
**Relative mRNA and protein levels of MMPs and MMPs inhibitors in MDA-MB-231 cells treated with TGF-β1 and a p38 MAPK inhibitor**. MDA-MB-231 cells were pre-treated for 1 h with different concentrations (0, 5, 10 or 20 μM) of SB203680 (p38 MAPK pharmacological inhibitor) and then stimulated with 10 ng/mL TGF-β1 by 20 h. Total RNA from these samples was used to analyze the mRNA expression levels of MMPs (MMP-2 and MMP-9) and MMPs inhibitors (TIMP-2 and RECK) by qRT-PCR. The levels of pro-enzyme and active MMP-2 and MMP-9 proteins were evaluated by zymography. Total protein lysates were used to measure the protein levels of RECK by Western blotting. GAPDH protein was used as the loading control in Western blotting assays. Conditioned medium from these cultures were also used to analyze the TIMP-2 protein levels by Western blotting. Results are presented in graphics as means ± standard errors from three independent experiments. The Western blotting figures are representative of one experiment. For mRNA expression levels fold change: MMP-2 (a versus c: *p *< 0.05, c versus f: *p *< 0.05), MMP-9 (a versus c: *p *< 0.001, a versus d: *p *< 0.05, c versus f: *p *< 0.01), TIMP-2 (c versus e: *p *< 0.05, c versus f: *p *< 0.001) and RECK (a versus c: *p *< 0.01, c versus e: *p *< 0.05, c versus f: *p *< 0.001). For protein expression levels fold change: MMP-2 (a versus c: *p *< 0.01, c versus f: *p *< 0.05), MMP-9 (a versus c: *p *< 0.001, a versus d: *p *< 0.05), TIMP-2 (a versus c: *p *< 0.05, c versus f: *p *< 0.05) and RECK (a versus c, e, f: *p *< 0.001, a versus d: *p *< 0.05).

The role of p38 MAPK in the proposed TGF-β1-mediated mechanism was also investigated. MDA-MB-231 cells were pre-treated for 1 h with 0, 5, 10 or 20 μM of SB203680 (a p38 MAPK pharmacological inhibitor) followed by treatment with TGF-β1 (10 ng/mL). Inhibition of p38 MAPK pathway significantly blocked (*p *< 0.05) the TGF-β1-induced upregulation of MMP-2, MMP-9, TIMP-2 and RECK mRNA levels. Interestingly, lower concentrations of p38 MAPK inhibitor were required to abrogate the action of TGF-β1 on mRNA levels of MMPs inhibitors (TIMP-2 and RECK) (Figure 7).

The highest SB203680 concentration tested was able to significantly (*p *< 0.05) inhibit the TGF-β1-mediated induction of the active MMP-2 and TIMP-2 protein levels (Figure 7). On the other hand, inhibition of p38 MAPK did not have a significant effect on MMP-9 protein induction or RECK protein downregulation promoted by TGF-β1 treatment. Together, these data led us to propose that p38 MAPK was responsible for the mediation of the TGF-β1 effect on the MMP-2 and TIMP-2 protein levels. It is important to note that unlike ERK1/2 pathway, p38 MAPK activity was not relevant for the TGF-β1 modulation of MMP-9 and RECK expression.

### ERK1/2 and p38 MAPK pathways crosstalk in the MDA-MB-231 cellular model

The above results indicated that ERK1/2 and p38 MAPK pathways were involved in the TGF-β1-mediated regulation of MMPs and their inhibitors. Therefore, we investigated whether these signal transduction molecules could crosstalk in MDA-MB-231 cells upon activation by TGF-β1. To this end, MDA-MB-231 cells were pre-treated with 20 μM of an ERK1/2 or p38 MAPK inhibitor (PD98059 and SB203680, respectively) for 1 h and then stimulated with 10 ng/mL of TGF-β1. Since ERK1/2 and p38 MAPK displayed a different activation kinetics, upon the cellular pre-treatment with PD98059 or SB203680, we performed TGF-β1 stimulation for periods of times corresponding to the maximal activation of each MAPK observed in the previous experiments (Figure 5). Therefore, in addition to TGF-β1, cells were treated with ERK1/2 inhibitor for 10 min and 3 h and with the SB203680 for 30 min and 1 h.

**Figure 8:**
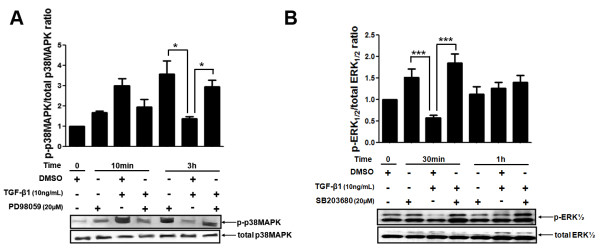
**Phosphorylated and total protein expression levels of p38 MAPK and ERK1/2 in MDA-MB-231 cell line treated with specific inhibitors for these MAPKs and stimulated with TGF-β1 for different periods of time**. (A) MDA-MB-231 cells were pre-treated with 20 μM of PD98059 (specific ERK1/2 pharmacological inhibitor) for 1 h and then stimulated with 10 ng/mL TGF-β1 for different periods of time (0, 10 min and 3 h). Total protein lysates from these samples were used to analyze the protein expression levels of total and phosphorylated forms of p38 MAPK by Western blotting. (B) The phosphorylated and total protein expression levels of ERK 1/2 were measured by Western blotting in MDA-MB-231 cells pre-treated with 20 μM of SB203680 (p38 MAPK pharmacological specific inhibitor) for 1 h and stimulated with 10 ng/mL TGF-β1 for 0, 30 min and 1 h. These results were analyzed and used to calculate the p-p38 MAPK/total p38 MAPK and p-ERK1/2/total ERK1/2 ratios, respectively. Results are presented in graphics as mean ± standard errors from three independent experiments. The Western blotting figures are representative of one experiment. *, *p *< 0.05, *** and *p *< 0.001, all versus control (cells treated with DMSO, vehicle).

TGF-β1 stimulation of MDA-MB-231 cells for 3 h did not affect p38 MAPK activation (Figure 8A). However, the levels of p-p38 MAPK were significantly higher (*p *< 0.05) in cells pre-treated with PD98059 relative to cells treated only with TGF-β1 for the longest period of time. Addition of TGF-β1 did not induce a significant change on p-p38MAPK accumulation in ERK 1/2 inhibited cells (Figure 8A). However, treatment with SB203680 promoted a similar effect on p-ERK1/2 levels for 30 min of treatment (Figure 8B). TGF-β1-treated cells had significantly lower p-ERK1/2 protein (*p *< 0.001) when compared with MDA-MB-231 cells pre-treated with the p38 MAPK specific inhibitor (Figure 8B). These results suggest that the ERK1/2 and p38 MAPK pathways crosstalk in the MDA-MB-231 cell model. However, TGF-β1 was apparently not involved in this signalling interaction.

### TGF-β1-increased migration and invasion capacities of MDA-MB-231 cells are dependent on ERK1/2, p38 MAPK and MMPs activities

**Figure 9:**
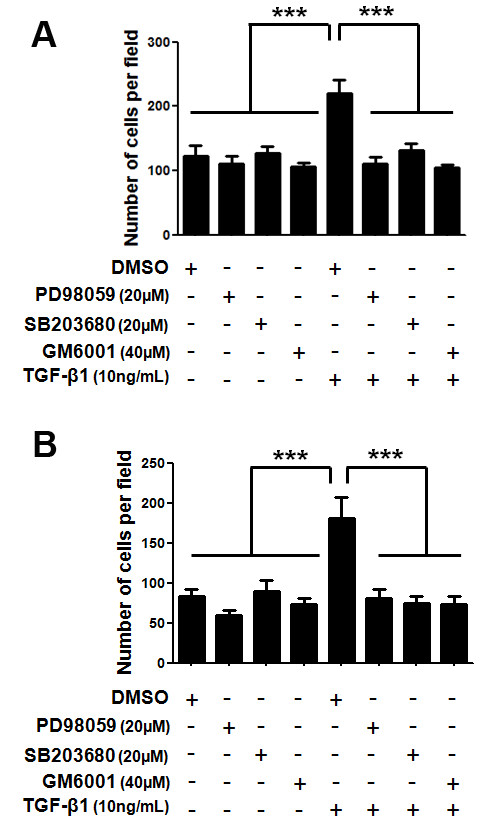
***In vitro *migration (A) and invasion (B) capacities of the MDA-MB-231 cell line upon treatment with TGF-β1 and inhibitors of ERK1/2, p38 MAPK and MMPs**. MDA-MB-231 cells were pre-treated for 1 h with 20 μM of either PD98059 or SB203680 (ERK1/2 and p38 MAPK pharmacological inhibitor, respectively) or with 40 μM of GM6001 (a broad-spectrum MMPs inhibitor). After the inhibition treatment these cells were stimulated with 10 ng/mL TGF-β1 and allowed to migrate through uncoated transwells for 8 h (A) or invade through matrigel-coated transwells for 24 h (B). The number of cells at the bottom of the transwell filters was counted at the end of each assay. Results are presented as means ± standard errors from three independent experiments, performed in duplicate, all versus control (cells treated with DMSO, vehicle).

Our results support the hypothesis that TGF-β1 is a common regulator of molecules classically related to cell motility and invasive phenotype. Thus, we examined the effect of this cytokine on the migratory and invasive potential of MDA-MB-231 cells. TGF-β1-treated MDA-MB-231 cells presented a significantly increased (*p *< 0.001) migration (Figure 9A) and invasion (Figure 9B) capacities, doubling the number of cells present at the bottom of transwells. Moreover, we investigated whether ERK1/2, p38 MAPK and MMPs could act as mediators of this TGF-β1-mediated effect in MDA-MB-231 motility. To this end, cells were pre-treated for 1 h with 20 μM of either PD98059 or SB203680, or with 40 μM of GM6001 (a broad-spectrum MMPs inhibitor), and then stimulated with 10 ng/mL TGF-β1. Treatment of the MDA-MB-231 cell line only with ERK1/2, p38 MAPK or MMPs inhibitors did not have a significant effect in the migratory and invasive phenotype in relation to cells treated with vehicle (DMSO). However, all of these inhibitors were able to significantly block (*p *< 0.001) the TGF-β1-induced migration and invasion potential of MDA-MB-231 cells, suggesting that TGF-β1 indeed utilizes ERK1/2 and p38 MAPK to mediate the upregulation of MMPs.

## Discussion

Metastasis is the final stage in tumor progression, being the main factor associated with cancer-promoted deaths [42]. The balance between the activities of MMPs and MMP inhibitors is the essential regulator of ECM degradation and, consequently, of cellular phenotypes related to motile and invasive capacities. Similar to other cancer types, the breast cancer progression process is positively correlated with increased MMPs and MMP inhibitors expression and activity [12], suggesting a coordinate regulation mechanism. In this report, we demonstrated, for the first time, that TGF-β1 is able to modulate MMP, TIMP and RECK expression in MDA-MB-231 human breast cancer cell line through ERK1/2 and p38MAPK. Both of these transducer pathways were essential to the TGF-β1-enhanced migration and invasion phenotypes; however, each mediated the TGF-β1 signal for MMPs and their inhibitors in a specific manner.

The important role of TGF-β during multiple stages of cancer progression has been widely reported. However, the status of several members of this pathway in human cancers remains quite complex and unclear [43,44]. The TGF-β receptors and their downstream transducers are frequently lost, mutated or attenuated in human carcinomas, including pancreatic, colon and gastric tumors [43,44]. Alternatively, other tumor types, such as breast tumors, present much lower mutation frequency in these TGF-β signaling effectors, but display many alterations in their expression levels [43,45,46]. Only few reports addressed more than one TGF-β pathway member at the same time. Due to the lack of information regarding profile complexity of the TGF-β network elements and their dependence on the cell context, we first performed a general characterization of the TGF-β isoforms and their receptors by mRNA expression analysis in a panel of five human breast cancer cell lines displaying diverse invasive and metastatic capacities. We showed that, similar to MMPs, TIMPs and RECK, the mRNA levels of TGF-β receptors I and II, are expressed at a higher level in the most aggressive cell line, as compared to the less invasive ones, except for TβRI that was also highly expressed in ZR-75-1 cells. These results corroborate prior reports in the literature from tumor tissue samples, showing that, in breast cancer models, TGF-β signaling appears to be correlated with tumor-promoting functions [23,47,48].

TGF-β1 acts as a growth inhibitor at the early stages of tumorigenesis while it stimulates EMT, tumor invasion and metastasis in advanced tumors [40,41]. Therefore, cancer cells in different stages of aggressiveness respond differently to TGF-β treatment. The least invasive (MCF-7) and the highly invasive (MDA-MB-231) human breast cancer cell lines are examples of this dual role of TGF-β. In this case, loss of estrogen receptor expression and *ras *gene amplification, two very common alterations during breast cancer progression, are some factors involved in switching the phenotypic response of TGF-β treatment, from anti-proliferative to invasive [32]. Thus, TGF-β1 is not able to regulate proliferation of the MDA-MB-231 cells [28,49]. However, we demonstrate that this cytokine is a positive modulator of migration and invasive potential of these cells.

Previous reports have suggested a crucial function of TGF-β1 in cell motility control, some of which relate this altered phenotype to its role as a modulator of MMPs [23-27,50]. Kim and collaborators suggested that TGF-β1 also induces invasion in pre-malignant breast cancer cells (MCF10A), by upregulation of MMP-2 and MMP-9 [21]. Subsequent reports also indicated that MMP-2 and MMP-9 are essential in the TGF-β1-incresead invasion of MCF10 cell series in a 3D model [23]. Similarly, the high motility phenotype presented by TGF-β1-treated MDA-MB-231 cells was associated with the upregulation of MMP-9 by this cytokine [50,51]. On the other hand, in the MDA-MB-435 cell line, MMP-14 was shown to be the molecule responsible for the TGF-β1-increased migration capacity [22]. However, none of these previous reports investigated whether TGF-β1 can also modulate the expression of MMP inhibitors, and whether these inhibitors, thought to downmodulate ECM breakdown, are also implicated in the TGF-β1-induced cell spreading. Since the balance between MMPs and their inhibitors is an important factor for ECM degradation, the identification of common regulators of MMPs, TIMPs and RECK is necessary to identify the principal factors involved in the metastatic process. Here we describe, for the first time, a molecular mechanism in which TGF-β1 modulates MMP-2 and MMP-9 as well as TIMP-2 and RECK expression. The regulation of these MMPs inhibitors expression could be related to a cellular response for reestablishment of the proteases/inhibitors balance during cancer progression.

We found some discrepancy between the mRNA and protein expression levels of some MMPs and MMPs inhibitors upon treatment with TGF-β1. For instance, while RECK was increased at the transcriptional level, its protein expression levels were inhibited by this cytokine. This divergence could be due to the influence of TGF-β1 in RECK mRNA and protein stability and degradation rates and/or to other post-transcriptional and post-translational molecular mechanisms.

Although mounting evidence supports the potential role of RECK as a molecular marker for cancer prognosis and controller of cellular metastatic capacity, no reports are available unveiling its function in breast cancer [18,19]. For the first time, we have demonstrated that expression of this membrane-associated MMP inhibitor is regulated by TGF-β1 in a breast cancer cell culture model, suggesting that RECK could be involved in the molecular mechanisms of breast cancer progression.

TGF-β1 is able to signal through both Smad-dependent and Smad-independent mechanisms. However, previous evidences have established that each of these pathways is related to distinct cellular responses to TGF-β1 [35,52]. Therefore, the switching of TGF-β's role from a tumor suppressor to a pro-oncogenic-factor during cancer progression could be caused by changes in the way that this cytokine modulates its downstream pathways. It has been suggested that Smads are involved in the anti-tumor process, such as inhibition of cell proliferation, while the Smad-independent pathways have been implicated in induction of tumor progression [35,52].

**Figure 10:**
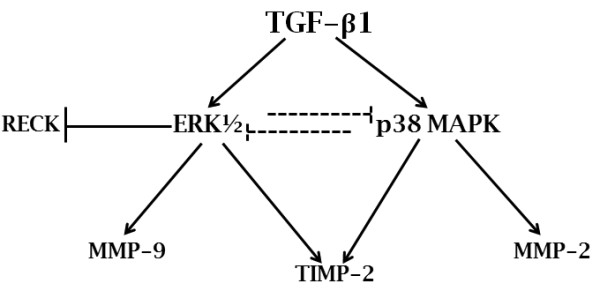
**Scheme of the molecular mechanism proposed for TGF-β1 action as a common regulator of MMPs (MMP-2 and MMP-9) and their inhibitors (TIMP-2 and RECK), through ERK1/2 and p38 MAPK pathways, in MDA-MB-231 cell line**. The dashed lines indicate regulations suggested by preliminary experiments.

Here we analyzed the involvement of ERK1/2 and p38 MAPK, two well established Smad-independent pathways, in the proposed mechanism of coordinate regulation of MMPs, TIMPs and RECK by TGF-β1 in breast cancer cell lines. Our results demonstrate that both MAPKs are important for this mechanism, each being responsible for modulating specific molecules (Figure 10). Unlike previously reported data of MCF10A cells [21], p38 MAPK as well as ERK1/2 were shown to be key components mediating the TGF-β1-induced MMPs upregulation. However, our data show that p38 MAPK mediates increased levels of MMP-2 and ERK1/2 are involved in the modulation of MMP-9 levels. Although both p38 MAPK and ERK1/2 were required for TGF-β1 induction of the TIMP-2 protein expression, we demonstrated that only ERK1/2 are responsible for the RECK downregulation induced upon TGF-β1 treatment [21].

## Conclusions

Taken together, the results obtained demonstrate that TGF-β1 is a common regulator of MMPs (MMP-2 and MMP-9) and their inhibitors (TIMP-2 and RECK) in breast cancer cell models. Besides TGF-β1 function in controlling extracellular matrix components synthesis [53], our results provide important evidence that this cytokine performs a central and intricate function in the control of the ECM status by the modulation of MMPs, TIMPs and RECK expression. Subsequent *in vivo *assays should be performed to further support our data. The TGF-β1-mediated balance among these proteases and their specific inhibitors seems to be a result of the equilibrium between p38 MAPK and ERK1/2 activities (Figure 10). The crosstalk between the MAPK pathways shown here could also increase the complexity of this TGF-β1 effect on cancer cells. Furthermore, the dose-dependent TGF-β1 functions on MMP-9 and RECK protein levels emphasize the multifaceted mechanism of this cytokine in the control of tumor invasion and metastatic capacities. Thus, the promising application of clinical approaches based on TGF-β1 targeting for breast cancer treatment may be very challenging, due to the complex and broad-spectrum actions of this cytokine in cancer progression and microenvironment architecture.

## Abbreviations

ECM: Extracellular matrix; EMT: Epithelial-mesenchymal transition; ER: Estrogen receptor; ERK: Extracellular signal-regulated kinase 1/2; MAPK: Mitogen-activated protein kinase; MMP: Matrix metalloproteinases; p-ERK: Phosphorilated form of extracellular signal-regulated kinase 1/2; p-p38 MAPK: Phosphorilated form of p38 MAPK; PR: Progesterone receptor; qRT-PCR: Quantitative reverse transcription polymerase chain reaction; RECK: Reversion-inducing cysteine-rich protein with Kazal motifs; TGF-β1: Transforming growth factor-β; TIMP: Tissue inhibitors of matrix metalloproteinases; TβR: Receptor of Transforming growth factor-β.

## Competing interests

The authors declare that they have no competing interests.

## Authors' contributions

LRG was responsible for most of the experimental work and results interpretation. LRG was also responsible for manuscript preparation. LFT and RAMW performed some experimental work. LL participated in the study design, data discussion and interpretation. MCS was involved in the study design and supervision. All authors read and approved the final manuscript.

## Pre-publication history

The pre-publication history for this paper can be accessed here:

http://www.biomedcentral.com/1471-2407/12/26/prepub

## Supplementary Material

Additional file 1 **Analysis of the relative expression levels of PAI-I as a positive control for MDA-MB-231 cell line treatment with (A) recombinant TGF-β1 or (B) TGF-β1 bioactivity neutralizing antibody**. The mRNA expression of PAI-I was analyzed by qRT-PCR using total RNA from the MDA-MB-231 cells treated with (A) 0, 1, 5 or 10 ng/mL of recombinant TGF-β1 for 20 h or (B) 0, 1, 10, 25 or 50 ng/mL of anti-TGF-β1 antibody for 24 h. The results are presented as means ± standard errors from two independent experiments. **, *p *< 0.01 and *** *p *< 0.001, all versus control (untreated cell). Click here for file
