# Supplementary figures and images for: TGF-β1 modulates the homeostasis between MMPs and MMP inhibitors through p38 MAPK and ERK1/2 in highly invasive breast cancer cells
Source: BMC Cancer. 2012 Jan 19;12:26. doi: 10.1186/1471-2407-12-26 (PMC3277461; doi:10.1186/1471-2407-12-26)

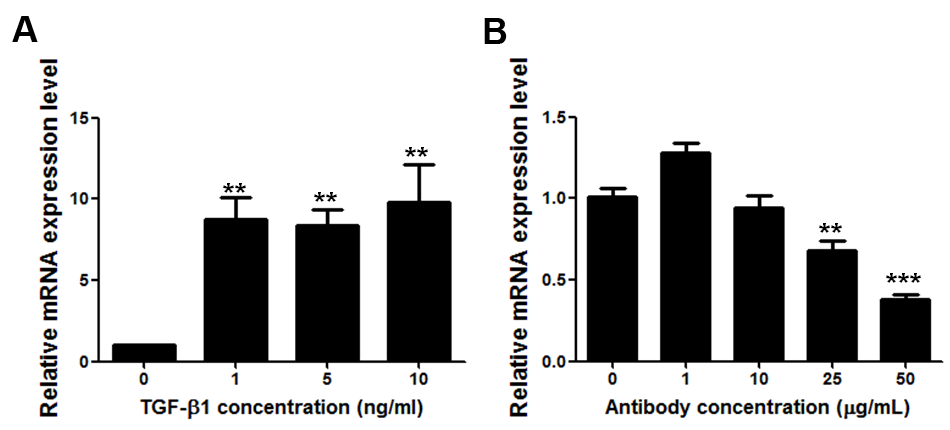

Supplement: Additional file 1 — Analysis of the relative expression levels of PAI-I as a positive control for MDA-MB-231 cell line treatment with (A) recombinant TGF-β1 or (B) TGF-β1 bioactivity neutralizing antibody. The mRNA expression of PAI-I was analyzed by qRT-PCR using total RNA from the MDA-MB-231 cells treated with (A) 0, 1, 5 or 10 ng/mL of recombinant TGF-β1 for 20 h or (B) 0, 1, 10, 25 or 50 ng/mL of anti-TGF-β1 antibody for 24 h. The results are presented as means ± standard errors from two independent experiments. **, p < 0.01 and *** p < 0.001, all versus control (untreated cell). [file 1471-2407-12-26-S1.TIFF]
